# FUNCTIONAL INDEPENDENCE, ACCESS TO KIDNEY TRANSPLANTATION, AND WAITLIST MORTALITY

**DOI:** 10.1093/geroni/igz038.1834

**Published:** 2019-11-08

**Authors:** Nadia M Chu, Stephanie Sison, Abimereki Muzaale, Christine Haugen, Jacqueline Garonzik Wang, Silas Norman, Dorry Segev, Mara McAdams-DeMarco

**Affiliations:** 1 Department of Surgery, Johns Hopkins School of Medicine, Baltimore, Maryland, United States; 2 Department of Medicine, Johns Hopkins University School of Medicine, Baltimore, Maryland, United States; 3 Department of Surgery, Johns Hopkins School of Medicine, Maryland, Baltimore, United States; 4 Department of Medicine, Division of Nephrology, University of Michigan, Ann Arbor, Michigan, United States; 5 Department of Epidemiology, Johns Hopkins Bloomberg School of Public Health, Baltimore, Maryland, United States

## Abstract

Although functional independence is a health priority for patients with advanced CKD, 50% of those who progress to end-stage kidney disease (ESKD) develop difficulties carrying-out essential day-to-day activities. Functional independence is not routinely assessed at kidney transplant (KT) evaluation; therefore, it is unclear what percentage of candidates are functionally independent and whether independence is associated with access to KT and waitlist mortality. We studied a prospective cohort of 3,168 ESKD participants (1/2009-6/2018) who self-reported functional independence in basic Activities of Daily Living (ADL) and more complex Instrumental Activities of Daily Living (IADL). We estimated adjusted associations between functional independence (separately) and listing (Cox), waitlist mortality (competing risks), and transplant rates (Poisson). At evaluation, 92.4% were independent in ADLs, but only 68.5% were independent in IADLs. Functionally independent participants had a higher chance of listing for KT (ADL:aHR=1.55,95%CI:1.30-1.87; IADL:aHR=1.39,95%CI 1.26-1.52). Among KT candidates, ADL independence was associated with lower waitlist mortality risk (SHR=0.66,95%CI:0.44-0.98) and higher rate of KT (IRR=1.58,95%CI:1.12-2.22); the same was not observed for IADL independence (SHR=0.86,95%CI:0.65-1.12; IRR=1.01,95%CI:0.97-1.19). ADL independence was associated with better KT access and lower waitlist mortality; clinicians should screen KT candidates for ADL independence, and identify interventions to maintain independence to improve waitlist outcomes.

